# Deep learning based sentiment analysis and offensive language identification on multilingual code-mixed data

**DOI:** 10.1038/s41598-022-26092-3

**Published:** 2022-12-13

**Authors:** Kogilavani Shanmugavadivel, V. E. Sathishkumar, Sandhiya Raja, T. Bheema Lingaiah, S. Neelakandan, Malliga Subramanian

**Affiliations:** 1 Department of Artificial Intelligence, Kongu Engineering College, Perundurai, Erode, 638060 India; 2 Department of Industrial Engineering, Hanyang University, 222 Wangsimini-ro, Seongdong-gu, Seoul, 04763 Republic of Korea; 3 Department of Information Technology, Kongu Engineering College, Perundurai, Erode, 638060 India; 4 Departmemt of Biomedical Engineering, Jimma Institute of Technology, Jimma, Ethiopia; 5 Department of Computer Science and Engineering, R.M.K Engineering College, Chennai, Tamilnadu India; 6 Department of Computer Science and Engineering, Kongu Engineering College, ERODE, 638060 India

**Keywords:** Engineering, Mathematics and computing

## Abstract

Sentiment analysis is a process in Natural Language Processing that involves detecting and classifying emotions in texts. The emotion is focused on a specific thing, an object, an incident, or an individual. Although some tasks are concerned with detecting the existence of emotion in text, others are concerned with finding the polarities of the text, which is classified as positive, negative, or neutral. The task of determining whether a comment contains inappropriate text that affects either individual or group is called offensive language identification. The existing research has concentrated more on sentiment analysis and offensive language identification in a monolingual data set than code-mixed data. Code-mixed data is framed by combining words and phrases from two or more distinct languages in a single text. It is quite challenging to identify emotion or offensive terms in the comments since noise exists in code-mixed data. The majority of advancements in hostile language detection and sentiment analysis are made on monolingual data for languages with high resource requirements. The proposed system attempts to perform both sentiment analysis and offensive language identification for low resource code-mixed data in Tamil and English using machine learning, deep learning and pre-trained models like BERT, RoBERTa and adapter-BERT. The dataset utilized for this research work is taken from a shared task on Multi task learning DravidianLangTech@ACL2022. Another challenge addressed by this work is the extraction of semantically meaningful information from code-mixed data using word embedding. The result represents an adapter-BERT model gives a better accuracy of 65% for sentiment analysis and 79% for offensive language identification when compared with other trained models.

## Introduction

Now-A-days, using the internet to communicate with others and to obtain information is necessary and usual process. The majority of people may now use social media to broaden their interactions and connections worldwide. Persons can express any sentiment about anything uploaded by people on social media sites like Facebook, YouTube, and Twitter in any language. Pattern recognition and machine learning methods have recently been utilized in most of the Natural Language Processing (NLP) applications^1^. Each day, we are challenged with texts containing a wide range of insults and harsh language. Automatic intelligent software that detects flames or other offensive words would be beneficial and could save users time and effort. These works defy language conventions by being written in a spoken style, which makes them casual. Because of the expanding volume of data and regular users, the NLP has recently focused on understanding social media content^2^.

The number of social media users is fast growing since it is simple to use, create and share photographs and videos, even among people who are not good with technology. Many websites allow users to leave opinions on non-textual information such as movies, images and animations. YouTube is the most popular of them all, with millions of videos uploaded by users and billions of opinions. Detecting sentiment polarity on social media, particularly YouTube, is difficult. Deep learning and other transfer learning models help to analyze the presence of sentiment in texts. However, when two languages are mixed, the data contains elements of each in a structurally intelligible way. Because code-mixed information does not belong to a single language and is frequently written in Roman script, typical sentiment analysis methods cannot be used to determine its polarity^3^.

The approach of extracting emotion and polarization from text is known as Sentiment Analysis (SA). SA is one of the most important studies for analyzing a person's feelings and views. It is the most well-known task of natural language since it is important to acquire people's opinions, which has a variety of commercial applications. In recent years, SA in social media has risen in popularity. SA is a text mining technique that automatically analyzes text for the author's sentiment using NLP techniques^4^. The goal of SA is to identify the emotive direction of user evaluations automatically. The demand for sentiment analysis is growing as the need for evaluating and organizing hidden information in unstructured way of data grows. Offensive Language Identification (OLI) aims to control and minimize inappropriate content on social media using natural language processing. On media platforms, objectionable content and the number of users from many nations and cultures have increased rapidly. In addition, a considerable amount of controversial content is directed toward specific individuals and minority and ethnic communities. As a result, identifying and categorizing various types of offensive language is becoming increasingly important^5^.

The rise in increasing popularity of social media has led to a surge in trolling, hostile and insulting comments, which really is a significant problem in terms of the good and bad effects that a communication can have on a person or group of people. Offensive language is any text that contains specific types of improper language, such as insults, threats, or foul phrases. This problem has prompted various researchers to work on spotting inappropriate communication on social media sites in order to filter data and encourage positivism. Hate speech appears to be identical from foul words in comparison. The earlier seeks to identify 'exploitative' sentences, which are regarded as a kind of degradation^6^.

Offensive language can be discovered in a number of different ways. To prevent cyberbullying, a supervised learning technique was applied, which was focused on three key factors: contents, online bullying, and user-based features^7^. This research work focused on analyzing sentiment in YouTube comments using various learning models. The data is code-mixed social networking data taken from YouTube comments provided by ACL2022 shared task. In keeping with the qualities of social media, the text is informal and conversational. The text is first preprocessed to normalize the unfamiliar words like punctuation, stop words, HTML tags, emojis and changing all the sentences to lower case^8^.

In recent years, classification of sentiment analysis in text is proposed by many researchers using different models, such as identifying sentiments in code-mixed data^9^ using an auto-regressive XLNet model. The accuracies obtained for both datasets are 49% and 35%, respectively. Despite the fact that the Tamil-English mixed dataset has more samples, the model is better on the Malayalam-English dataset; this is due to greater noise in the Tamil-English dataset, which results in poor performance. These results can be improved further by training the model for additional epochs with text preprocessing steps that includes oversampling and undersampling of the minority and majority classes, respectively^10^.

An embedding is a learned text representation in which words with related meanings are represented similarly. The most significant benefit of embedding is that they improve generalization performance particularly if you don't have a lot of training data. GloVe is an acronym that stands for Global Vectors for Word Representation. It is a Stanford-developed unsupervised learning system for producing word embedding from a corpus's global phrase co-occurrence matrix. The essential objective behind the GloVe embedding is to use statistics to derive the link or semantic relationship between the words. The proposed system adopts this GloVe embedding for deep learning and pre-trained models. Another pretrained word embedding BERT is also utilized to improve the accuracy of the models.

The organization of the paper is as follows: “Related work” section discusses the related work done by other authors for identifying sentiments and offensive languages. “Proposed system” section @@describes the dataset, preprocessing techniques that are used and the proposed methodology. The results and evaluation measures are discussed in “Performance evaluation” section. Finally, the proposed work is concluded and future work is outlined in “Conclusion” section.

## Related work

Some authors recently explored with code-mixed language to identify sentiments and offensive contents in the text. Code-mixed languages include multiple languages in the same dataset. Sentiment analysis of code-mixed comments on social media in three common Dravidian languages, including Tamil, Kannada, and Malayalam, using pre-trained models like ULMFiT and multilingual BERT fine-tuned on the code-mixed dataset, transliteration (TRAI), English translations (TRAA), and a combination of all the three^11^ highlights the Dravidian-work Code-mixed data significance at FIRE 2021.On TRAI and TRAA, the F1 scores for ULMFiT were nearly equal, at 65.8% and 65.1%, respectively. Similar results were obtained using ULMFiT trained on all four datasets, with TRAI scoring the highest at 70%. For the identical assignment, BERT trained on TRAI received a competitive score of 69%. At FIRE 2021, the results were given to Dravidian Code-Mix, where the top models finished in the fourth, fifth, and tenth positions for the Tamil, Kannada, and Malayalam challenges.

Tamil-English and Malayalam-English are two Dravidian languages for which sentiment analysis has been proposed^12^. The Logistic Regression classifier is used in conjunction with pre-trained models like BERT, DistilBERT, and fasttext. The f1 score for the Tamil-English dataset was 0.58, whereas the f1 score for the Malayalam-English dataset was 0.63. When the findings were presented to the Dravidian Code-Mix FIRE 2020, the Tamil-English language pair scored 8/14, while the Malayalam-English language pair scored 11/15. Positive, negative, mixed, neutral, and not in intended language are the class labels. These models include BERT, DistilBERT, and XLM-RoBERTa, which are pre-trained transformer models. Kannada-English obtained the F1 score of 0.630, Malayalam-English got the F1 score of 0.804, and Tamil-English achieved an F1 score of 0.711.The results are presented to the Dravidian code-mixed shared task held at FIRE 2021^13^.

The Dravidian Code-Mix-FIRE 2020 has been informed of the sentiment polarity of code-mixed languages like Tamil-English and Malayalam-English^14^. Pre-trained models like the XLM-RoBERTa method are used for the identification. It also uses the k-fold method to solve sentiment analysis. The F1 score of Malayalam-English achieved 0.74 and for Tamil-English, the F1 score achieved was 0.64.

Identification of offensive language using transfer learning contributes the results to Offensive Language Identification in shared task on EACL 2021. The dataset contains code-mixed data’s with six different classes. The pretrained models like CNN + Bi-LSTM, mBERT, DistilmBERT, ALBERT, XLM-RoBERTa, ULMFIT are used for classifying offensive languages for Tamil, Kannada and Malayalam code-mixed datasets. Without doing preprocessing of texts, ULMFiT achieved massively good F1-scores of 0.96, 0.78 on Malayalam and Tamil, and DistilmBERT model achieved 0.72 on Kannada^15^.

Offensive language is identified by using a pretrained transformer BERT model^6^. This transformer recently achieved a great performance in Natural language processing. The datasets were taken from the German eval shared tasks2 context. Due to an absence of models that have already been trained in German, BERT is used to identify offensive language in German-language texts has so far failed. This BERT model is fine-tuned using 12 GB of German literature in this work for identifying offensive language. This model passes benchmarks by a large margin and earns 76% of global F1 score on coarse-grained classification, 51% for fine-grained classification, and 73% for implicit and explicit classification.

In^16^, the authors worked on the BERT model to identify Arabic offensive language. The effects of transfer learning are investigated across different Arabic offensive language datasets in this study and constructed numerous classifiers with mix of four datasets to gather information about online Arabic offensive content and classify user comments accordingly. The findings show that transfer learning is used across individual datasets from different sources and themes, such as YouTube comments from musician’s channels and Aljazeera News comments from political stories, yields unsatisfactory results. Overall, the results of the experiments show that need of generating new strategies for pre-training the BERT model for Arabic offensive language identification.

Sentiment analysis is performed on Tamil code-mixed data by capturing local and global features using machine learning, deep learning, transfer learning and hybrid models^17^. Out of all these models, hybrid deep learning model CNN + BiLSTM works well to perform sentiment analysis with an accuracy of 66%. In^18^, aspect based sentiment analysis known as SentiPrompt which utilizes sentiment knowledge enhanced prompts to tune the language model. This methodology is used for triplet extraction, pair extraction and aspect term extraction.

Empirical study was performed on prompt-based sentiment analysis and emotion detection^19^ in order to understand the bias towards pre-trained models applied for affective computing. The findings suggest that the number of label classes, emotional label-word selections, prompt templates and positions, and the word forms of emotion lexicons are factors that biased the pre-trained models^20^.

Affective computing and sentiment analysis^21^ can be exploited for affective tutoring and affective entertainment or for troll filtering and spam detection in online social communication. This work discusses about the way for the development of more bioinspired approaches to the design of intelligent sentiment-mining systems that can handle semantic knowledge, make analogies, learn new affective knowledge, and detect, perceive, and “feel” emotions. In^20^, the authors proposed commonsense-based neurosymbolic framework that employed unsupervised and reproducible subsymbolic techniques such as auto-regressive language models and kernel methods to build trustworthy symbolic representations that convert natural language to a sort of protolanguage and, hence, extract polarity from text in a completely interpretable and explainable manner^22,23^.

The existing system with task, dataset language, and models applied and F1-score are explained in Table 1.

**Table 1:** Existing systems.

References	Tasks	Language	Model	F1 score
Ou and Li^14^	Sentiment analysis	Malayalam–English	XLM-RoBERTa	0.74
		Tamil–English	XLM-RoBERTa	0.63
Mahata et al.^32^	Sentiment analysis	English–Tamil	Bi-LSTM	0.61
			LSTM	0.62
Banerjee et al.^9^	Sentiment analysis	Tamil–English	XL-Net	0.52
		Malayalam–English	XL-Net	0.32
Chanda and Pal^12^	Sentiment analysis	Tamil–English	BERT Distil BERT Fasttext	0.58 0.57 0.58
		Malayalam–English	BERT Distil BERT Fasttext	0.60 0.61 0.63
Varma et al.^33^	Sentiment analysis	Telungu–English	LR NB SVM RF NN	0.76 0.67 0.74 0.75 0.78
Saumya et al.^34^	Offensive language identification	Malayalam code–mixed	BERT ULMFit	0.62 0.52
		Tamil code–mixed	BERT ULMFit	0.86 0.76
Hande et al.^35^	Offensive language identification	Tamil	mBERT XLM-R DistilmBERT MUTiL IndicBERT ULMFiT	0.75 0.61 0.74 0.61 0.72 0.78
		Malayalam	mBERT XLM-R DistilmBERT MUTiL IndicBERT ULMFiT	0.93 0.92 0.94 0.82 0.95 0.96
		Kannada	mBERT XLM-R DistilmBERT MUTiL IndicBERT ULMFiT	0.69 0.68 0.70 0.38 0.68 0.70

## Proposed system

The datasets using in this research work available from^24^ but restrictions apply to the availability of these data and so not publicly available. Data are however available from the authors upon reasonable request and with permission of^24^. It is split into a training set which consists of 32,604 tweets, validation set consists of 4076 tweets and test set consists of 4076 tweets. The dataset contains two features namely text and corresponding class labels. The class labels of sentiment analysis are positive, negative, Mixed-Feelings and unknown State. The total number of texts in each category is illustrated in the Table 2.

**Table 2:** Sentiment analysis class labels details.

Class labels	Training data comments	Test data comments
Positive	19,533	4076
Negative	4034	
Mixed-feelings	3828	
Unknown state	5209	
Total	32,604	

In positive class labels, an individual's emotion is expressed in the sentence as happy, admiring, peaceful, and forgiving. The language conveys a clear or implicit hint that the speaker is depressed, angry, nervous, or violent in some way is presented in negative class labels. Mixed-Feelings are indicated by perceiving both positive and negative emotions, either explicitly or implicitly. Finally, an unknown state label is used to denote the text that is unable to predict either as positive or negative^25^.

The class labels of offensive language are not offensive, offensive targeted insult individual, offensive untargeted, offensive targeted insult group and offensive targeted insult other. The total number of texts in each category is represented in Table 3.

**Table 3:** Offensive language identification class labels details.

Class labels	Training data comments	Test data comments
Not offensive	24,523	4076
Offensive targeted insult group	2463	
Offensive targeted insult other	469	
Offensive targeted insult individual	2308	
Offensive untargeted	2841	
Total	32,604	

Not offensive class label considers the comments in which there is no violence or abuse in it. Without a specific target, the comment comprises offense or violence then it is denoted by the class label Offensive untargeted. These are remarks of using offensive language that isn't directed at anyone in particular. Offensive targeted individuals are used to denote the offense or violence in the comment that is directed towards the individual. Offensive targeted group is the offense or violence in the comment that is directed towards the group. Offensive targeted other is offense or violence in the comment that does not fit into either of the above categories^8^.

### Word embedding

An embedding is a learned text representation in which words with related meanings are represented similarly. GloVe is an acronym that stands for Global Vectors for Word Representation. It's a Stanford-developed unsupervised learning system for producing word embedding from a corpus's global phrase co-occurrence matrix. The essential objective behind the GloVe embedding is to use statistics to derive the link between the words. The Embedding method is used to boost the accuracy of the models^26,27^. Google's BERT is a new method for obtaining pre-trained word vectors. BERT can take one or two sentences as input and differentiate them using the special token [SEP]. The [CLS] token, which is unique to classification tasks, always appears at the beginning of the text^17^.

### BERT and GLOVE embedding

As BERT uses a different input segmentation, it cannot use GloVe embeddings. GloVe uses simple phrase tokens, whereas BERT separates input into sub—word parts known as word-pieces. In any case, BERT understands its configurable word-piece embeddings along with the overall model. Because they are only common word fragments, they cannot possess its same type of semantics as word2vec or GloVe^21^.

### Methodology

**Figure 1:**
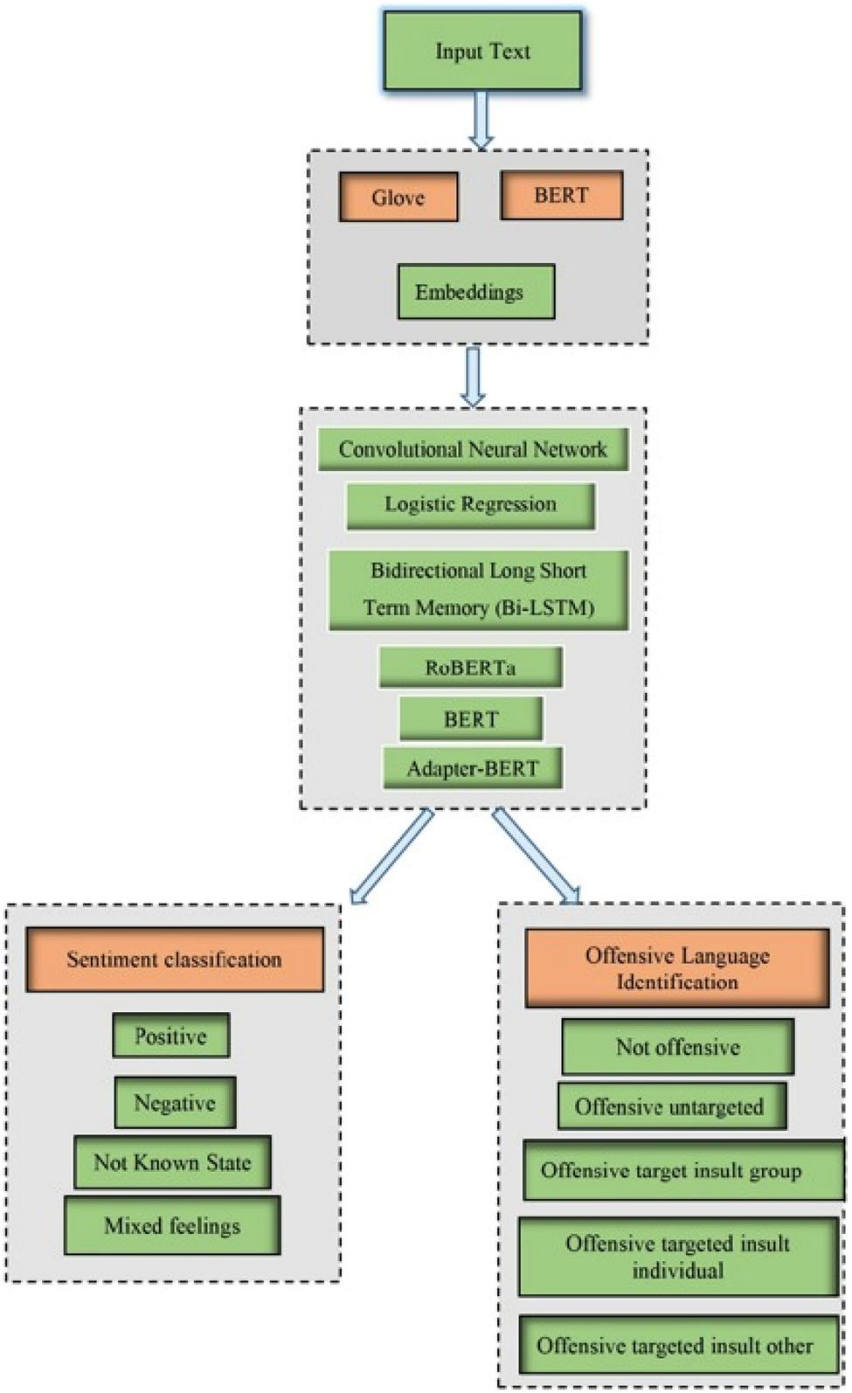
Proposed system structure.

The process of concentrating on one task at a time generates significantly larger quality output more rapidly. In the proposed system, the task of sentiment analysis and offensive language identification is processed separately by using different trained models. Figure 1 illustrates the process of the proposed system. It covers the overall functionalities of all models. A code-mixed text dataset with total of 4076 comments are given as input. These comments are taken from the shared task of ACL anthology. Different machine learning and deep learning models are used to perform sentimental analysis and offensive language identification. To get accurate predictions, the text is preprocessed. Preprocessing steps include removing stop words, changing text to lowercase, and removing emojis. These preprocessing are done only for machine learning models. After preprocessing the text, Glove and Bert embeddings is used. These embeddings are used to represent words and works better for pretrained deep learning models. The Embeddings also boosts the accuracy of the models. Embeddings encode the meaning of the word such that words that are close in the vector space are expected to have similar meanings. By training the models, it produces accurate classifications and while validating the dataset it prevents the model from overfitting and is performed by dividing the dataset into train, test and validation. The set of instances used to learn to match the parameters is known as training. Validation is a sequence of instances used to fine-tune a classifier's parameters. The texts are learned and validated for 50 iterations, and test data predictions are generated. These steps are performed separately for sentiment analysis and offensive language identification. The pretrained models like Logistic regression, CNN, BERT, RoBERTa, Bi-LSTM and Adapter-Bert are used text classification. The classification of sentiment analysis includes several states like positive, negative, Mixed Feelings and unknown state. Similarly for offensive language identification the states include not-offensive, offensive untargeted, offensive targeted insult group, offensive targeted insult individual and offensive targeted insult other. Finally, the results are classified into respective states and the models are evaluated using performance metrics like precision, recall, accuracy and f1 score.

#### Logistic regression

Logistic regression is a classification technique and it is far more straightforward to apply than other approaches, specifically in the area of machine learning. Also, it works well when the dataset can be separated linearly.

#### Convolutional neural network

The CNN has pooling layers and is sophisticated because it provides a standard architecture for transforming variable-length words and sentences of fixed length distributed vectors. For sentence categorization, we utilize a minimal CNN convolutional network, however one channel is used to keep things simple. To begin, the sentence is converted into a matrix, with word vector representations in the rows of each word matrix. The weight matrix is used to parameterize a filter. To obtain a length n vector from a convolution layer, a 1-max pooling function is employed per feature map. The final categorization is obtained using a softmax algorithm. Finally, dropouts are used as a regularization method at the softmax layer^28,29^.

#### RoBERTa

Robustly Optimized BERT Pre-training Approach is known as RoBERTa. Although RoBERTa's architecture is essentially identical to that of BERT, it was designed to enhance BERT's performance. This suggests that RoBERTa has more parameters than the BERT models, with 123 million features for RoBERTa basic and 354 million for RoBERTa wide^30^.

#### Bidirectional long short term memory

A recurrent neural network used largely for natural language processing is the bidirectional LSTM. It may use data from both sides and, unlike regular LSTM, input passes in both directions. Furthermore, it is an effective tool for simulating the bidirectional interdependence between words and expressions in the sequence, both in the forward and backward directions. The outputs from the two LSTM layers are then merged using a variety of methods, including average, sum, multiplication, and concatenation. Bi-LSTM trains two separate LSTMs in different directions (one for forward and the other for backward) on the input pattern, then merges the results^28,31^. Once the learning model has been developed using the training data, it must be tested with previously unknown data. This data is known as test data, and it is used to assess the effectiveness of the algorithm as well as to alter or optimize it for better outcomes. It is the subset of training dataset that is used to evaluate a final model accurately. The test dataset is used after determining the bias value and weight of the model. The dataset is then applied to the test data. Accuracy obtained is an approximation of the neural network model's overall accuracy^23^.

#### BERT

Bidirectional Encoder Representations from Transformers is abbreviated as BERT. It is intended to train bidirectional LSTM characterizations from textual data by conditioning on both the left and right context at the same time. As an outcome, BERT is fine-tuned just with one supplemental output layer to produce cutting-edge models for a variety of NLP tasks^20,21^.

#### Adapter-BERT

**Figure 2:**
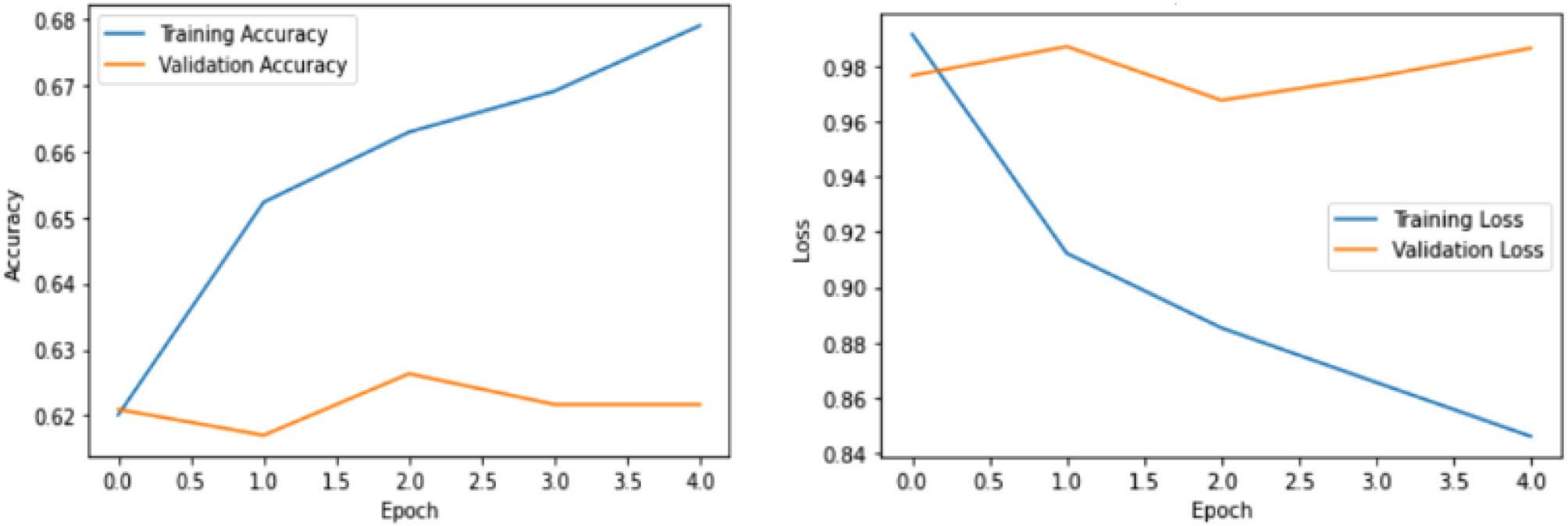
Training and validation accuracy and loss values for sentiment analysis task using adapter-BERT.

Adapter-BERT inserts a two-layer fully-connected network that is adapter into each transformer layer of BERT. Only the adapters and connected layer are trained during the end-task training; no other BERT parameters are altered, which is good for CL and since fine-tuning BERT causes serious occurrence. Adapter-BERT outperforms fine-tuned BERT in terms of performance. Figure 2 illustrates the architecture of adapter-BERT^17,18^.

## Performance evaluation

There are different methods for assessing the effectiveness of the model. Precision, Recall, Accuracy and F1-score are the metrics considered for evaluating different deep learning techniques used in this work. The metrics are derived as follows.

The proportion of positive cases that were accurately predicted is known as precision and is derived in the Eq. (1).

1 $$Precision = True\,Positive/(True\,Positive + {\text{False}}\,{\text{Positive}})$$

The proportion of correctly identified positive instances is known as recall and is derived in the Eq. (2).

2 $$Recal = True\,Positive/\left( {True\,Positive + False\,Negative} \right)$$

Precision and recall's harmonic mean is known as the F1-score and is derived in the Eq. (3).

3 $${F1-Score} = 2*Precision*Recall/Precision + Recall$$

In the total amount of predictions, the proportion of accurate predictions is called accuracy and is derived in the Eq. (4).

4 $$Accuracy = \left( {True\,Positive + True\,Negative} \right)/ \left( {True\,Positive + False\,Positive + True\,Negative + False\,Negative} \right)$$

From Tables 4 and 5, it is observed that the proposed Bi-LSTM model for identifying sentiments and offensive language, performs better for Tamil-English dataset with higher accuracy of 62% and 73% respectively.

**Table 4:** Classification report of different models for sentiment analysis.

Class label	Measures	Logistic regression	CNN	Bi-LSTM	RoBERTa	BERT	Adapter-BERT
Positive	Precision	0.42	0.62	0.05	0.22	0.23	0.38
	Recall	0.37	0.80	0.23	0.28	0.20	0.25
	F1-Score	0.39	0.69	0.09	0.24	0.21	0.31
	Support	614	2379	109	469	469	469
Negative	Precision	0.66	0.17	0.24	0.37	0.41	0.40
	Recall	0.76	0.08	0.46	0.37	0.36	0.39
	F1-Score	0.71	0.11	0.32	0.37	0.39	0.43
	Support	2058	542	282	542	542	542
Mixed_Feelings	Precision	0.27	0.28	0.92	0.74	0.73	0.82
	Recall	0.21	0.18	0.66	0.69	0.77	0.78
	F1-Score	0.24	0.22	0.77	0.71	0.75	0.73
	Support	601	686	3287	2379	2379	2379
Unknown_State	Precision	0.47	0.15	0.29	0.40	0.46	0.46
	Recall	0.40	0.09	0.51	0.42	0.45	0.39
	F1-Score	0.43	0.11	0.37	0.41	0.46	0.44
	Support	803	469	398	686	686	686

**Table 5:** Classification report of different models for offensive language identification.

Class label	Measures	Logistic regression	CNN	Bi-LSTM	RoBERTa	BERT	Adapter-BERT
Not offensive	Precision	0.82	0.76	0.95	0.91	0.88	0.89
	Recall	0.90	0.92	0.83	0.73	0.84	0.86
	F1-Score	0.85	0.83	0.88	0.81	0.86	0.88
	Support	2775	3049	3487	3049	3049	3049
Offensive targeted insult group	Precision	0.36	0.11	0.13	0.19	0.27	0.17
	Recall	0.29	0.05	0.17	0.41	0.41	0.38
	F1-Score	0.32	0.07	0.15	0.26	0.33	0.29
	Support	384	302	222	302	302	302
Offensive targeted insult individual	Precision	0.42	0.12	0.11	0.30	0.33	0.43
	Recall	0.30	0.05	0.12	0.39	0.35	0.38
	F1-Score	0.35	0.07	0.12	0.34	0.34	0.32
	Support	392	283	270	283	283	283
Offensive targeted insult other	Precision	0.00	0.00	0.00	0.06	0.00	0.00
	Recall	0.00	0.00	0.00	0.15	0.00	0.00
	F1-Score	0.00	0.00	0.00	0.09	0.00	0.00
	Support	34	48	0	48	48	48
Offensive untargeted	Precision	0.40	0.23	0.06	0.37	0.42	0.44
	Recall	0.32	0.08	0.23	0.49	0.41	0.43
	F1 Score	0.36	0.11	0.09	0.42	0.41	0.44
	Support	491	394	97	394	394	394

Figure 2 shows the training and validation set accuracy and loss values using Bi-LSTM model for sentiment analysis. From the figure it is observed that training accuracy increases and loss decreases. So, the model performs well for sentiment analysis when compared to other pre-trained models.

**Figure 3:**
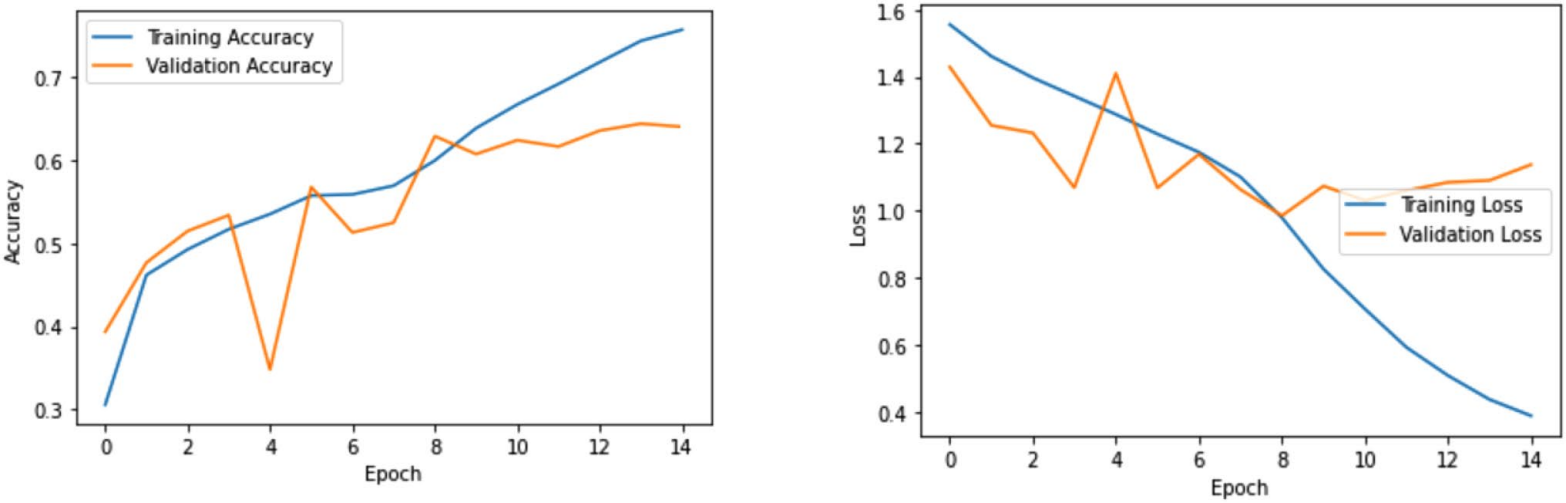
Training and validation accuracy and loss values for offensive language identification using adapter-BERT.

Figure 3 shows the training and validation set accuracy and loss values of Bi-LSTM model for offensive language classification. From the figure, it is observed that training accuracy increases and loss decreases. So, the model performs well for offensive language identification compared to other pre-trained models.

**Figure 4:**
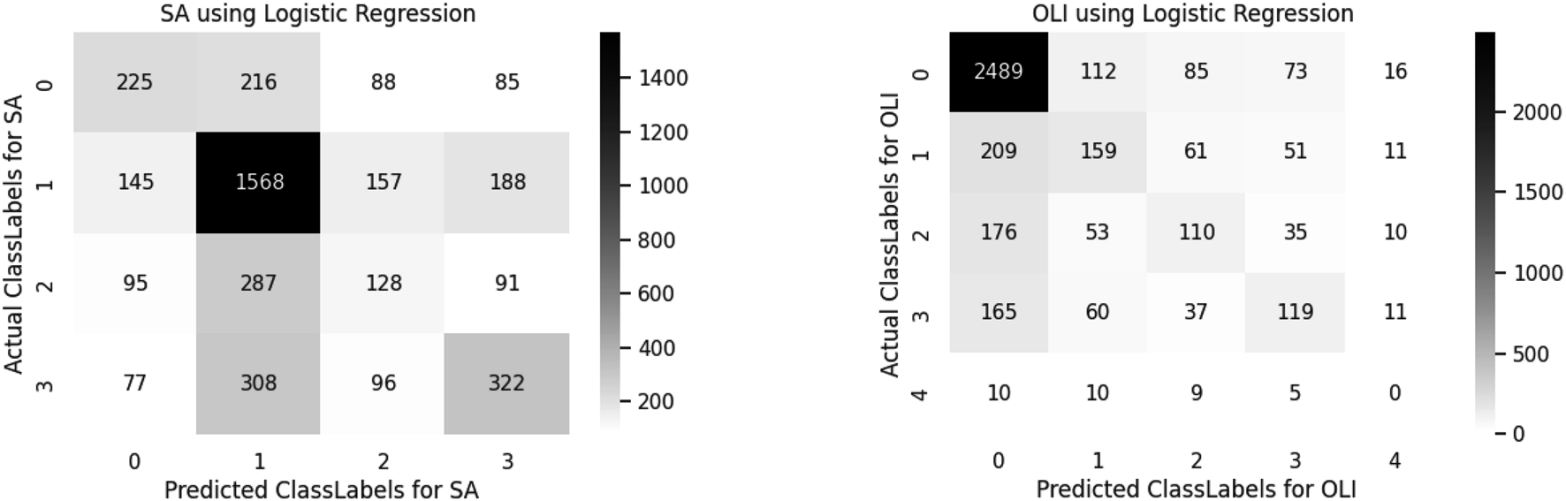
Confusion matrix of logistic regression for sentiment analysis and offensive language identification.

**Figure 5:**
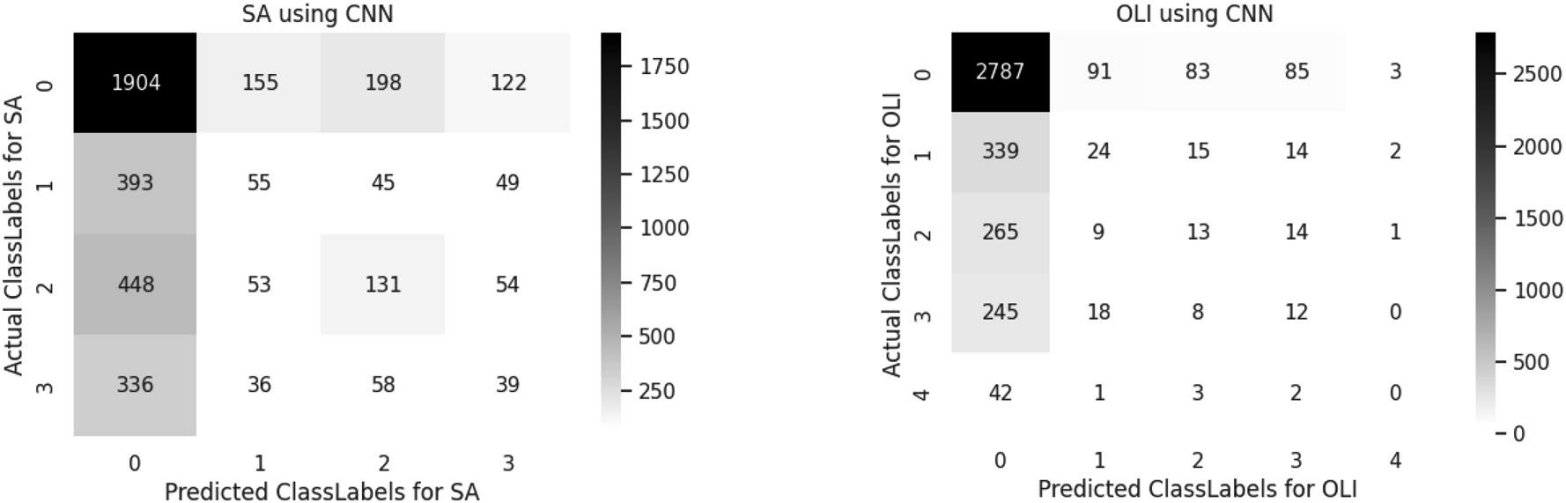
Confusion matrix of CNN for sentiment analysis and offensive language identification.

**Figure 6:**
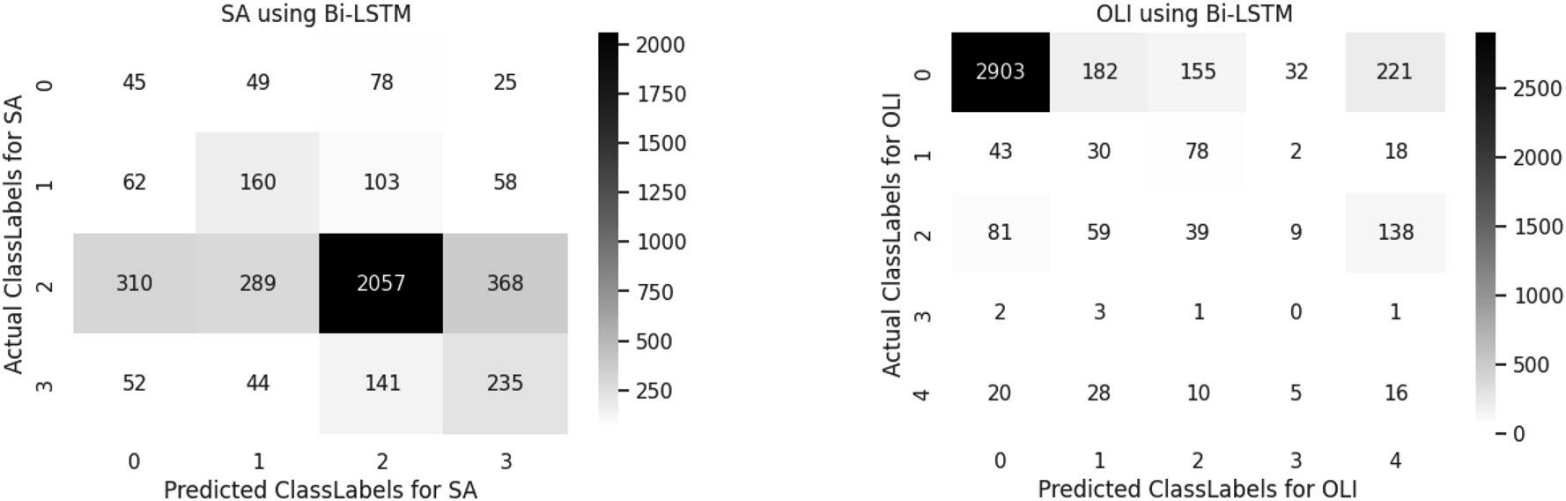
Confusion matrix of Bi-LSTM for sentiment analysis and offensive language identification.

**Figure 7:**
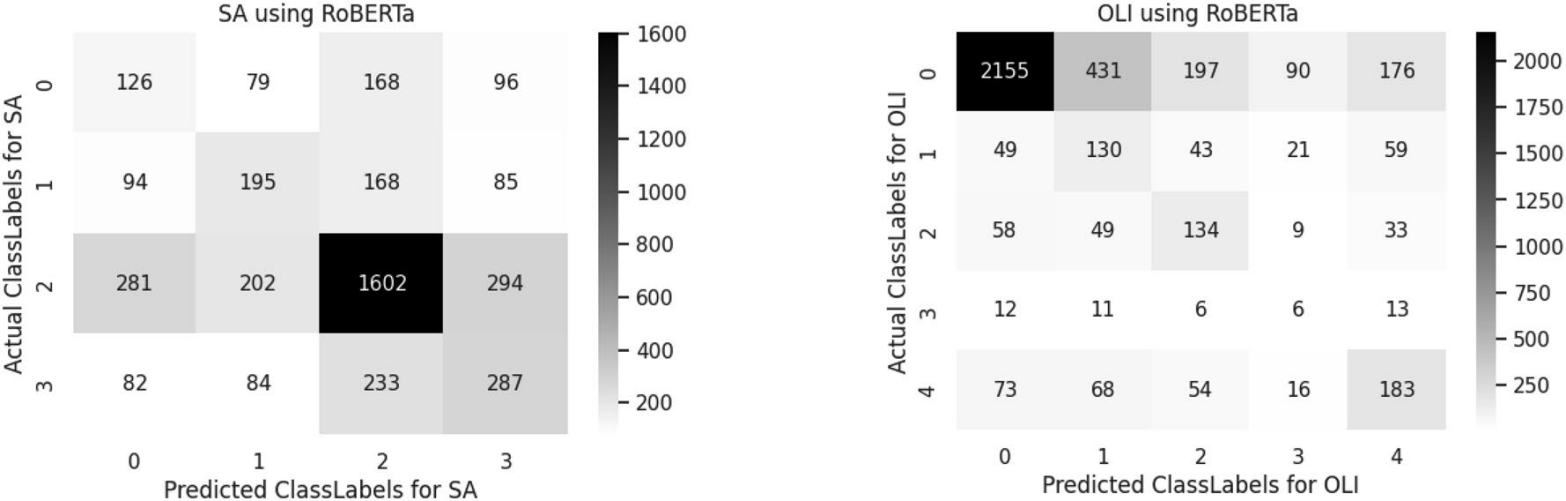
Confusion matrix of RoBERTa for sentiment analysis and offensive language identification.

**Figure 8:**
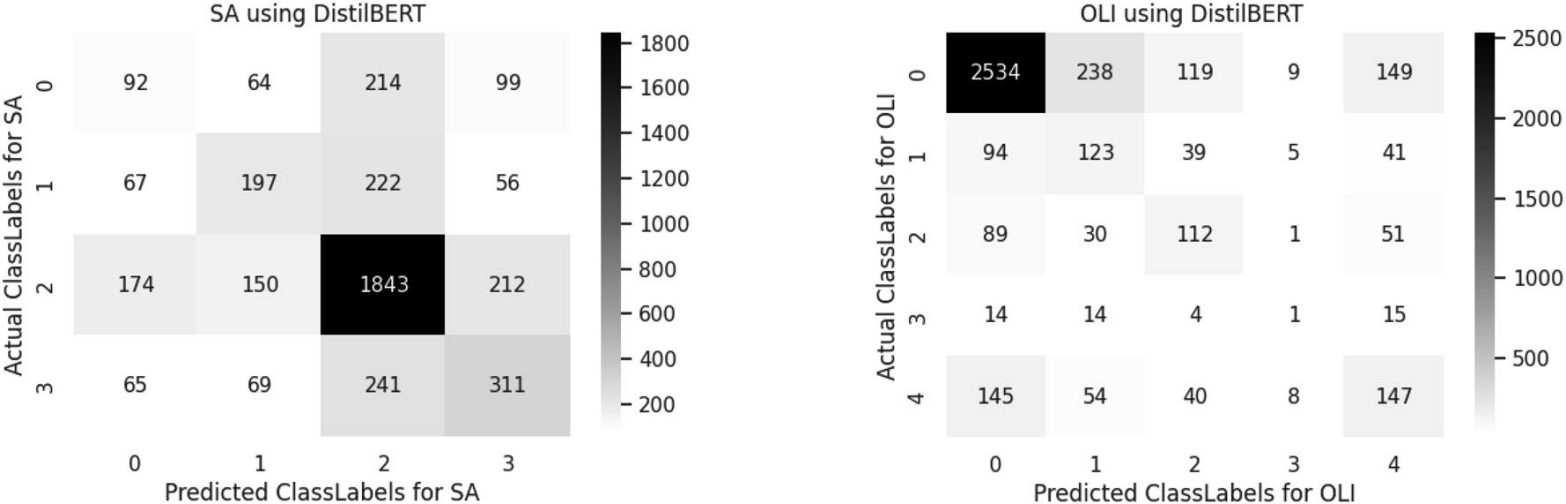
Confusion matrix of BERT for sentiment analysis and offensive language identification.

**Figure 9:**
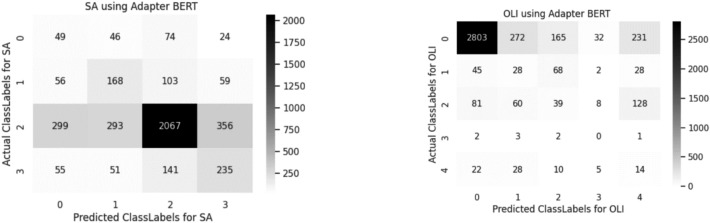
Confusion matrix of adapter-BERT for sentiment analysis and offensive language identification.

A confusion matrix is used to determine and visualize the efficiency of algorithms. The confusion matrix of both sentiment analysis and offensive language identification is described in the below Figs. 4, 5, 6, 7, 8 and 9. The class labels 0 denotes positive, 1 denotes negative, 2 denotes mixed feelings, and 3 denotes an unknown state in sentiment analysis. Similarly, in offensive language identification, the class labels are 0 denotes not offensive, 1 denotes offensive untargeted, 2 denotes offensive targeted insult group, 3 denotes offensive target insult individual, and 4 denotes offensive target insult other.

Logistic regression predicts 1568 correctly identified negative comments in sentiment analysis and 2489 correctly identified positive comments in offensive language identification. The confusion matrix obtained for sentiment analysis and offensive language Identification is illustrated in the Fig. 4.

CNN predicts 1904 correctly identified positive comments in sentiment analysis and 2707 correctly identified positive comments in offensive language identification. The confusion matrix obtained for sentiment analysis and offensive language Identification is illustrated in the Fig. 5.

Bidirectional LSTM predicts 2057 correctly identified mixed feelings comments in sentiment analysis and 2903 correctly identified positive comments in offensive language identification. The confusion matrix obtained for sentiment analysis and offensive language Identification is illustrated in the Fig. 6.

RoBERTa predicts 1602 correctly identified mixed feelings comments in sentiment analysis and 2155 correctly identified positive comments in offensive language identification. The confusion matrix obtained for sentiment analysis and offensive language identification is illustrated in the Fig. 7.

BERT predicts 1043 correctly identified mixed feelings comments in sentiment analysis and 2534 correctly identified positive comments in offensive language identification. The confusion matrix is obtained for sentiment analysis and offensive language Identification is illustrated in the Fig. 8.

Confusion matrix for Adapter-BERT is illustrated in the Fig. 9. From the above obtained results Adapter-BERT performs better for both sentiment analysis and Offensive Language Identification. As Adapter-BERT inserts a two layer fully connected network in each transformer layer of BERT.

### Error analysis

This section analyses the performance of proposed models in both sentiment analysis and offensive language identification system by examining actual class labels with predicted one. The first sentence is an example of a Positive class label in which the model gets predicted correctly. The same is followed for all the classes such as positive, negative, mixed feelings and unknown state. Sample outputs from our sentiment analysis task are illustrated in Table 6.

**Table 6:** Error analysis of samples—sentiment analysis.

Example	Actual	Positive	Negative	Mixed feelings	Unknown state
Telugu thala fans hit likes	Positive	Yes	No	No	No
Vijay anna fans & veriyans hit like	Unknown state	Yes	No	No	No
Blockbuster aga valthukal from sk bloods	Negative	Yes	No	No	No

The proposed model Adapter-BERT correctly classifies the 1st sentence into the positive sentiment class. Next, consider the 2nd sentence, which belongs to the unknown state. It can be observed that the proposed model wrongly classifies it into the positive category. The reason for this misclassification may be because of the word “furious”, which the proposed model predicted as having a positive sentiment. If the model is trained based on not only words but also context, this misclassification can be avoided, and accuracy can be further improved. Similarly, the model classifies the 3rd sentence into the positive sentiment class where the actual class is negative based on the context present in the sentence. Table 7 represents sample output from offensive language identification task.

**Table 7:** Error analysis of samples—offensive language identification.

Example	Actual	Not offensive	Offensive targeted insult group	Offensive untargeted	Offensive targeted individual	Offensive targeted insult other
Ngk maass kaapaan teaser maasu maranam	Not offenisve	Yes	No	No	No	No
THILLALANGIDI part jr	Not Offensive	No	No	Yes	No	No
Inime vijay fansku zoomaka zoo than	Offensive Targeted Insult Individual	No	Yes	No	No	No
Akshay kumar ku bathila siva kumar ah potrukanum thappu pannitanga	Offensive Targeted Insult Other	No	No	No	No	Yes

The proposed Adapter-BERT model correctly classifies the 1st sentence into the not offensive class. Next, consider the 2nd sentence, which belongs to the not offensive class. It can be observed that the proposed model wrongly classifies it into the offensive untargeted category. The reason for this misclassification which the proposed model predicted as having a untargeted category. If the model is trained based on not only words but also context, this misclassification can be avoided, and accuracy can be further improved. Next, consider the 3rd sentence, which belongs to Offensive Targeted Insult Individual class. It can be observed that the proposed model wrongly classifies it into Offensive Targeted Insult Group class based on the context present in the sentence. The proposed Adapter-BERT model correctly classifies the 4th sentence into Offensive Targeted Insult Other.

## Conclusion

Language is an important way of communicating. On social media platforms like Twitter, Facebook, YouTube, etc., people are posting their opinions that have an impact on a lot of users. The comments that contain positive, negative and mixed feelings words are classified as sentiments and the comments that contain offensive and not offensive words are classified as offensive language identification. Identifying sentiments on social media, particularly YouTube, is difficult. Similarly identifying and categorizing various types of offensive language is becoming increasingly important. For identifying sentiments and offensive language different pretrained models like logistic regression, CNN, Bi-LSTM, BERT, RoBERTa and Adapter-BERT are used. Among the obtained results Adapter BERT performs better than other models with the accuracy of 65% for sentiment analysis and 79% for offensive language identification. In future, to increase system performance multitask learning can be used to identify sentiment analysis and offensive language identification.

## Data Availability

The datasets using in this research work available from^24^ but restrictions apply to the availability of these data and so not publicly available. Data are however available from the authors upon reasonable request and with permission of^24^.
